# Implementation of the electronic health record in the German healthcare system: an assessment of the current status and future development perspectives considering the potentials of health data utilisation by representatives of different stakeholder groups

**DOI:** 10.3389/frhs.2024.1370759

**Published:** 2024-05-10

**Authors:** Elisabeth Rau, Tim Tischendorf, Beate Mitzscherlich

**Affiliations:** Faculty of Health and Healthcare Sciences, University of Applied Sciences Zwickau, Zwickau, Saxony, Germany

**Keywords:** digital health, electronic health record (EHR), personal health records, health data use, digitalisation

## Abstract

**Introduction:**

The digitalisation of the German healthcare system enables a wide range of opportunities to utilize healthcare data. The implementation of the EHR in January 2021 was a significant step, but compared to other European countries, the implementation of the EHR in the German healthcare system is still at an early stage. The aim of this paper is to characterise the structural factors relating to the adoption of the EHR in more detail from the perspective of representatives of stakeholders working in the German healthcare system and to identify existing barriers to implementation and the need for change.

**Methods:**

Qualitative expert interviews were conducted with one representative from each of the stakeholder groups health insurance, pharmacies, healthcare research, EHR development and panel doctors.

**Results:**

The interviews with the various stakeholders revealed that the implementation process of the EHR is being delayed by a lack of a viable basis for decision-making, existing conflicts of interest and insufficient consideration of the needs of patients and service providers, among other things.

**Discussion:**

The current status of EHR implementation is due to deficiency in legal regulations as well as structural problems and the timing of the introduction. For instance, the access rights of various stakeholders to the EHR data and the procedure in the event of a technical failure of the telematics infrastructure are remain unclear. In addition, insufficient information and communication measures have not led to the desired acceptance of EHR use among patients and service providers.

## Introduction

In order to achieve comprehensive digitalisation of the German healthcare system, all stakeholders must agree to implement this change together. In addition to hospitals, doctors' surgeries and health insurance companies, patients and politicians are also involved. This change includes the creation of digital infrastructures on the one hand and the digitalisation of procedures and processes in healthcare on the other (1). The digitalisation of the healthcare system offers a multitude of opportunities. In the German healthcare system alone, there is an economic potential of 42 billion euros per year (2). Other potentials include opportunities to develop new diagnostic and treatment options, improve communication between individual players in the healthcare system and between patients and service providers. The quality of care can also become better (3). One basis for realising the aforementioned potential is the use of healthcare data generated in everyday care. To effectively use this data, a platform is required where it can be digitally collated and then made accessible to selected groups of people. In this context, the electronic health record is a central and promising application. The contents of the record include data on diagnostics and medical treatment in the doctor's surgery and in hospitals, as well as data on the supply of medicines or from providers of remedies and aids.

With regard to the current status of digitalisation in various healthcare systems, it is clear that Germany still much ground to cover compared to other countries. This is reflected, among other things, in the fact that the implementation of electronic health records in everyday care in the German healthcare system has not yet been realised to the desired extent. In Europe, Estonia and Denmark in particular have comprehensively digitalised their healthcare systems (4). In Denmark, for example, the EHR was introduced as early as 2012 and in Estonia as early as 2008. In addition, almost the entire population in both countries currently has an EHR (3). They use the digital data of the healthcare system and provide patients and authorised service providers with additional information from the consolidation and analysis of a wide variety of data, in particular through the use of data from electronic health records.

In order to promote the further development of the German healthcare system with regard to electronic health records, it is necessary to identify the causal factors that have caused the delay this process thus far. Consequently, these factors should be analysed in a way that allows adjustment of these factors with the aim of achieving the objectives.

### Definition of an electronic health record

The health record is a document to be kept by the service providers involved in the treatment of a patient. All essential medical data that arises during treatment must be documented in the record. This includes information from the medical history, the measures applied and their results, as well as diagnoses made by the service provider during the patient's treatment. The patient's explanations and consent are also recorded. A patient's entire medical history thus becomes part of the record (5).

In the context of this work, the term electronic health record refers to the integrative electronic health record for an electronic documentation system that can be viewed and edited by doctors and other service providers involved in the treatment across disciplines, institutions and sectors [(3), p. 70].

In the EHR consent procedure, a basic distinction is made between the opt-out and opt-in procedure. While in the opt-out procedure, an EHR is set up for each patient or insured person as long as they do not expressly object, in an opt-in procedure, patients or insured persons must actively contact a corresponding authority in order to open a file.

### Development and current status of implementation in the German healthcare system

The German healthcare system can be divided into three levels: the legal framework, self-administration and the individual players. The legal framework becomes the responsibility of the federal, state and local governments. The Federal Ministry of Health is in charge of health policy within the federal government. However, the organisation of healthcare is carried out by the self-administration. This means that the individual institutions organise themselves independently and thus guarantee the provision of medical care [(6), p. 18]. The individual stakeholders who are ultimately directly involved in patient care include the medical profession and various healthcare professionals, hospitals and pharmacies. To enable them to represent their interests at the health policy level, the stakeholders are organised in professional organisations, as well as professional and business associations (6). Around 84.3 million citizens need to be cared for within the German healthcare system (7). The healthcare system is primarily financed by statutory and private health insurance. These in turn are financed by the contributions of their members.

gematik is one of the central institutions involved in the digitalisation of the healthcare system. The Federal Ministry of Health holds the largest share in gematik with 51%. gematik's tasks primarily include the introduction, operation and further development of the telematics infrastructure, the electronic health card and associated specialised applications, as well as the creation of an interoperability directory. It also assumes responsibilities in the area of data security (8). With regard to health data protection, the Bundesbeauftragter für den Datenschutz und die Informationsfreiheit [Federal Commissioner for Data Protection and Information Security] (BfDI) is a key authority. This is the public supervisory authority for all public bodies of the federal government as well as for certain social security organisations. Issues relating to data protection and data security must be coordinated with the BfDI (9). Each of the 16 federal states in Germany also has its own state data protection officer and corresponding supervisory authorities.

Several laws form the legal framework for the development and design of digitalisation in the German healthcare system, particulary for the implementation of the EHR and use of healthcare data. In addition, the requirements of the Datenschutz-Grundverordnung [General Data Protection Regulation] (GDPR) and the Bundesdatenschutzgesetz [Federal Data Protection Act] (BDSG) apply in Germany. The basis for digitalisation in the German healthcare system became law with the Act for Secure Digital Communication and Applications in Healthcare (E-Health Act), which came into force on 29 December 2015.

The EHR was introduced as a patient-moderated record in the German healthcare system in January 2021. From this point onwards, anyone with statutory health insurance can obtain an EHR on request from their health insurance provider. Private health insurers can offer an EHR on a voluntary basis; therefore, there is no legal obligation. The term patient-moderated means that the patient alone decides whether and to what extent they use the record and to whom they make which data available. Data that can be entered into the EHRs includes care and service data. Patients can also enter their own health data, such as that collected by wearable devices, into the record (10–12). Access becomes possible via an app provided by the respective health insurance company using a smartphone, tablet or computer. The prerequisite for using the EHR is prior registration with the respective health insurance company. EHR registration currently takes place either via the new electronic health card with NFC interface and a PIN applied for the card or alternatively via two-factor authentication. As of April 2023, around 667,449 people with statutory health insurance, which corresponds to around 1% of people with statutory health insurance, have an EHR (13). In addition, the records that currently exist are hardly filled with data. Due to the low number of existing records, utilisation by service providers is also at a very low level. Finally, data is exchanged between service providers via the telematics infrastructure. Access to the telematics infrastructure is via a connector (14). The use of EHR data, for example, for research projects, should be possible from 2023 for a selected group of authorised applicants. In this context, patients should be given the opportunity to voluntarily make their data available for research projects as part of a data donation through an opt-in procedure. Until now, using the data contained in EHRs for research purposes or merging data collected at different locations in the healthcare system has not been possible.

Most recently, in March 2023, the Federal Ministry of Health presented its digitalisation strategy for the healthcare and nursing sectors up to 2030. In this context, gematik is to become a digital health agency and will be tasked with defining comprehensive binding requirements for interoperability. The further development of the lead data protection supervisory authority is also planned. The aim is to become a standardised data protection supervisory practice in the health and care sector. One of the objectives of the strategy is to facilitate access to pseudonymised health data for researchers. The decisive interface in this context is the research data centre, through which the data is to be made available after an application has been submitted. In addition, around 80% of people with statutory health insurance should have an EHR by 2025. These goals are to be achieved, among other things, with the legislative proposals presented in this context for a Health Data Utilisation Act and a Digital Act. As part of a digital law, the consent procedure for the EHR is to become an opt-out procedure (15).

The aim of this study is to scrutinise the structural factors in the context of EHR implementation from the perspective of stakeholders working in the German healthcare system and, based on this, to identify existing barriers to implementation and the need for change. The aim is to answer the question of how relevant actors in the German healthcare system assess the current structural conditions regarding the implementation of her. Additionally, the study aims to explore existing implementation hurdles or areas that require change related to this topic and examine the causes contributing to the current status of EHR implementation, particularly regarding the potential use of health data in the German healthcare system. The focus here will be on the current legal regulations and the framework conditions of the healthcare system.

## Methodological approach

The methodological approach was based on a qualitative research design. In view of the defined objectives of the work, the investigation using a qualitative method is suitable, as it offers the possibility of describing an object of investigation in detail and developing hypotheses and theories. The approach taken in the study is exploratory in character and is intended to provide initial insights into the research topic (16). In order to ensure the quality of this scientific work, the catalogue of criteria from Lincoln et al. (17) was used. Firstly, it is necessary to define individual specific subject areas in relation to the general survey topic. The specific interview topics were selected on the basis of expert opinions on the one hand and a selective literature review on the other. In addition to the access authorisations of various stakeholders to the data contained in the EHR, the topics also include the existence or expansion of corresponding infrastructures and technical requirements, data protection and information security as well as the needs of patients with regard to the design of the EHR. The development of the EHR since its introduction and the resulting potential are also discussed. With regard to the field of investigation, the decision was made to take a multi-perspective view of the various representatives of stakeholder groups in the German healthcare system (16). With regard to the existing structures in the German healthcare system, the central stakeholder groups can be identified as service providers, patients, health insurers, academic and other public research, developers and manufacturers as well as healthcare policy and authorities [(18), p. 30]. However, the analysis should be limited exclusively to the perspectives of healthcare providers, patients, health insurers, developers and researchers. The views of health policy makers and authorities will deliberately not be included in the study, as both actors have a controlling or monitoring function regarding the implementation of the EHR and the use of health data. Consequently, they are not directly involved in the utilisation. However, the focus should be on these actors who are directly involved in the utilisation. The selection of these stakeholders can be justified by the fact that they formulate stakeholder-specific requirements and needs for the use of the EHR in everyday care and work as well as for the design of the record itself. These requirements and needs play a crucial role in the design of the legal framework and the EHR itself, so that it can be integrated seamlessly into care provision. The sample was then compiled from the previously defined field of investigation. This should be as heterogeneous a group of people as possible, with maximum contrast in the relevant characteristics and therefore informative for the study (19). With regard to representative of the health insurance stakeholder group, the analysis should focus on the level of statutory health insurance. Statutory health insurers cover around 90% of the German population (20). In addition, they have become legally obliged to provide an EHR for their policyholders with corresponding requirements. Private health insurers, on the other hand, cover a comparatively smaller proportion of the population. In addition, there are no precisely defined requirements for the provision of EHRs. In order to comprehensively reflect the perspective of the statutory health insurance funds, it is necessary for them to be represented by a high-ranking representative from the management level of a nationally active statutory health insurance fund. In addition, the representative must have extensive professional experience and appropriate specialist knowledge in the context of the EHR and its development. The service provider perspective is to be illuminated by doctors on the one hand and pharmacies on the other. The selection of pharmacies is justified by the fact that they have the role of supplying medicines within the healthcare system. By utilising the health data contained in the EHR, they would become empowered to actively improve the quality of the supply of medicines to patients. Both the representative of the medical profession and the representative of the pharmacy profession must be a person who acts within the framework of one of the corresponding nationwide interest groups. Only on this basis can the results be generalised beyond the specific case. In addition, it is necessary for the physician and pharmacy representative to have extensive professional experience and expertise in the context of the EHR and its development. For the area of research, the analysis should be carried out from the perspective of health services research, as EHR data in particular forms a central basis for their activities (11). The developer and manufacturer perspective is to be presented by a member of an EHR development team from the statutory health insurance funds and the patient perspective by a patient representative from a nationwide patient organisation. These representatives of the relevant stakeholder group must also hold a high-ranking position in their professional activities, have extensive professional experience and appropriate knowledge of the EHR and its development. Recruitment was carried out through the relevant contact persons of a nationwide association of pharmacies, patients and statutory health insurance physicians. The contact details of two other people were obtained from the contact database available as part of the work. The identified potential interviewees were then contacted by email and the subject of the research and the preliminary interview guidelines were briefly described. Finally, the interviewees were selected on the basis of predefined criteria such as length of professional experience, existing knowledge of the EHR and its development, and professional position. The result was a sample of five male interviewees (Table 1). This is therefore a convenience sampling [(16), p. 306]. The interviewees were selected for the study because they were easy to reach and met the predefined inclusion criteria. In accordance with the aforementioned requirements for the participants, this is not a random sample.

**Table 1:** Composition of the sample.

ID	Professional field of activity	Professional experience	Duration of the interview
B1	Head of a business unit in a large statutory health insurance company in Germany; The responsibilities of the division include representing the interests of the health insurance fund at federal level	30 years in the healthcare sector; Head of division since 2015	17 min
B2	Pharmacist who works in the telematics department of a nationwide pharmacy association	24 years in the healthcare sector	31 min
B3	Researcher in the field of health services research; team leader of a research team of a large statutory health insurance company in Germany	20 years in the field of health services research	35 min
B4	Team leader of an EHR development team on behalf of a large statutory health insurance company in Germany	28 years with a large statutory health insurance company; Since 2016 with the topic of developing an EHR	33 min
B5	Economist who works in the telematics and digitalisation department of a nationwide association of statutory health insurance physicians	25 years in the healthcare sector With a nationwide association of statutory health insurance physicians since 2019	36 min

ID, identifier; Min, minutes.

The semi-structured interview method in the form of guided interviews was chosen to illustrate the structural conditions of EHR implementation from the perspective of the actors working in the German healthcare system. The reason for choosing this method is that the interviewees, who represent one of the previously defined actors, have a certain proximity to both the topic and the corresponding actor due to the nature of their work. They can describe their view of the situation subjectively. This variant of the guided interview is also referred to as an expert interview, as the interviewees are technical experts on a specific topic whose structural expertise is to be developed with the help of the interview [(16), p. 375]. The semi-structured interview is based on an interview guideline, which roughly specifies and structures the questions to be answered by the interviewee. The guideline itself is considered flexible. Accordingly, additional or in-depth questions that arise during the interview can be asked [(16), p. 358]. The data collection took place in the period from 1 March 2023 to 20 March 2023. The appointments were arranged in advance by email or in person. While one interview took place face-to-face, the other interviews were conducted as hybrid interviews. Before the interviews were conducted, all interviewees were informed about the use of the data and a verbal declaration of consent was obtained. In addition, all interviews were recorded. All interviews were based on the same interview guide.

The material obtained from the guided interviews was initially available in the form of audio recordings. These were then transcribed into a document with the help of AI-supported transcription software. Then the documents were then compared with the audio recordings and any content that was not correctly transferred by the software was corrected with the help of the audio recordings. The transcription became complete and was carried out without adapting the wording. Only dialect was not taken into account in this context and was translated into standardised German. Punctuation marks were used when revising the transcripts according to sound and not grammatical correctness. In addition, personal and company-related data became unrecognisable by replacing them with synonyms. Finally, the transcripts in Word documents were imported into the computer-aided analysis programme MAXQDA (version 2020). The data collected in the context of the interviews, which were subsequently transcribed, were available in qualitative form. This paper therefore uses the method of qualitative content analysis with the help of the computer-assisted analysis programme MAXQDA (Version 2020) [(16), p. 602]. With regard to the content analysis process model, the material was first analysed comprehensively based on the research question and text passages that deemed relevant were then marked. This was followed by the development of thematic main categories, which should roughly structure the material. These became deductively derived from the research question and the thematic complexes of the individual questions of the interview guide. Finally, the entire material became coded with the help of the main categories. In the next step, the text passages that were coded with the same main category were compiled. Subcategories were then inductively derived from the material within the individual main categories and the entire material was coded using the differentiated category system [(16), p. 557]. The final evaluation was carried out along the main categories. Only those categories that were classified as relevant to answering the research question were analysed. In addition, it was analysed whether there were correlations within a main category between the subcategories and between the main thematic categories. In addition, the statements of the interviewees were compared as part of the multi-perspective analysis.

## Results

In the following, the results of the evaluation of the qualitative interviews are presented along the main categories.

### Development of the EHR

The development of the EHR became predominantly negative for all respondents. In this context, four interviewees refer to the fact that only a very small number of people with statutory health insurance have an EHR. The representative of the statutory health insurance physicians cites the fact that it has never been defined what is meant by a patient-moderated record and how the use of the EHR is intended for service providers and in which architectures.

However, the EHR developer states that he considers the development of the EHR to be positive in terms of the functionality of the record.

### Access authorisations

The representative of the SHI-accredited physicians stated that a basis for the discussion on access authorisation must be created at the outset. This means that it must be determined for which group of people which information should be made accessible and for what reason.

In the context of access authorisations, three interviewees refer to the role of the record owners. The care researcher criticises the fact that the will of the record owners regarding the use of the data has not been sufficiently taken into account in the discussions on access authorisations to date. The representative of the SHI-accredited physicians emphasises the need to provide comprehensive information to record users on how the system works. Building on this basis, the record owners can ultimately become empowered to make informed decisions regarding access authorisations. The representative of the pharmacies is in favour of an opt-out procedure in order to give people who do not trust anonymisation the freedom to make their own decisions.

The EHR developer is in favour of access by all service providers involved in the treatment and justifies this with the fact that medicine depends on a holistic view of the state of health.

The representative of the health insurance company is in favour of health insurance companies having access and emphasises that they would thus become able to use the EHR data to support the insured person in their care. The representative of the pharmacies, on the other hand, emphasises that, in his view, the statutory health insurance funds should not be given access to the EHR data. The EHR developer states that if the health insurance funds are to be granted access, this must be regulated via an opt-in procedure, as in this case it is a matter of private interests. The EHR developer states that the release of data for research purposes should also be regulated via an opt-out objection procedure, as the data is used to further develop and improve medical care for the entire German population.

In this context, the representative of the health insurance company emphasised that research must be able to access the overall data of the healthcare system in anonymised form.

### Infrastructure and technical requirements

With regard to infrastructures and technical requirements, various problems were mentioned by the interviewees. The representative of the SHI-accredited physicians criticised the fact that the current discussion is mainly about fictitious usage scenarios. The processes and their process steps and how the relevant players are involved in these are not currently defined in relation to the infrastructures. However, these would become the basis for formulating requirements for corresponding architectures. The representative of the health insurance company points out that the different structures of the data from the outpatient and inpatient areas are seen as problematic from the health insurance company's perspective. This means that the data from the outpatient sector is transmitted quarterly and is therefore available to the health insurance funds with a considerable delay, which makes it difficult to react promptly to acute events. In this context, the pharmacy representative cites time pressure when introducing new applications as a problem. In the past, this mistake had already been made with e-prescriptions and applications had been introduced that were not yet fully developed for use in practice*.* The EHR developer emphasises that there would be an implementation problem in Germany. In his view, the providers of practice management systems or hospital information systems must become active and implement the specifications published by gematik in 2019. A functioning system would already exist and therefore no new concepts would be necessary.

All interviewees emphasised the necessity for technical requirements and infrastructures to ensure that data is input into the EHR in a structured manner. The representative of the SHI-accredited physicians emphasised that the various EHR usage scenarios must be defined at the outset. In addition, priorities must be set as to which clinical pictures and scenarios are currently most important in order to plan the further procedure for expanding the corresponding infrastructures on this basis. The representative of the health insurance company states that the system needs to be converted to the use of cloud applications. The representative of the pharmacies also stated that appropriate lead times must be guaranteed regarding to the technical requirements. In addition, future users would have to be trained in advance.

### Data protection and information security

In terms of data protection and information security, the EHR developer and the representative of the health insurance company consider the current design of the registration process for insured persons to be problematic. This is very complex, and it becomes an access barrier for users.

Two interviewees named specific requirements that they place on EHR use related to data protection and information security.

The representative of the statutory health insurance physicians points out that data protection and information security are a fundamental prerequisite for entry and should not be seen as a hurdle. The representative of the pharmacies highlights a further requirement that the possibility for patients to exercise their right to self-determination must be retained in the context of EHR use. In addition, the establishment of such a system should become possible in agreement with the BfDI.

### Patient requirements for the EHR

The representative of the pharmacies emphasises that the EHR must become simple and user-friendly. There must also be options for using a proxy, for example for people who do not have their own end device or those who cannot use the functions of the record themselves due to other restrictions.

The representatives of statutory health insurance physicians and pharmacies emphasise the need for comprehensive information and communication measures aimed at patients in order to create a basic understanding among them of what the aim of the record is and the aim of its use. All stakeholders involved in the record as well as the Federal Ministry of Health must become involved in information and communication.

The supply researcher stated that a basis of trust must be created among users. They must be able to trust that it is a functioning and secure application in terms of data protection, information security and user-orientation.

### Needs from the service provider perspective

The representative of the pharmacies addresses the needs from the perspective of the service providers and emphasises that additional costs incurred by the service providers through the use of the EHR should be remunerated. In this context, it must also be clarified which group of people checks that the records are up to date. In addition, the issue of liability for service providers must be clarified in advance. In particular, it must be clarified whether service providers are obliged to read all the data contained in the EHR. In addition, a regulation must be found on how to deal with technical problems or failures in day-to-day care.

### The challenges

Two interviewees commented on the challenges that they believe the German healthcare system will have to overcome in the context of the introduction. The representative of the health insurance company and the healthcare researcher point out that it will be challenging to bring together the various data sets and formats of health data to subsequently use them in the direct context of healthcare or to further develop healthcare from a scientific perspective. The healthcare researcher emphasises that a regulation must be found on how to deal with data that can be set by the patient themselves, such as vital signs data collected by smart watches. The health insurance company representative also sees a challenge in the mandatory filling of EHRs by service providers. With the introduction of a writing obligation, it would also be necessary to discuss corresponding sanctions for service providers in the event of non-compliance with the requirements

The health insurance company representative sees a further challenge in dealing with the current structure of data protection regulations. He emphasises that an assessment must be made of how important data protection is to the system and its stakeholders compared to the benefits of health data. This requires committees to carry out this assessment.

### Potential

The healthcare researcher, the health insurance company and pharmacy representatives emphasise the resulting benefits from an economic perspective for the entire German healthcare system. Due to the data situation, an optimisation of patient flows could be achieved. This would give practitioners the opportunity to deal with patients who actually fall within their area of specialisation. The pharmacy and health insurance company representatives emphasise that financial resources could be saved by avoiding incorrect treatment and duplicate examinations.

Respondents also mentioned improvements in the area of research and care. The EHR developer emphasises that improvements for research would be achieved through the availability of mass data on the health status of the entire German population. The German healthcare system would ultimately have to rely less on data from other countries. In this context, the representative of the statutory health insurance physicians criticised the fact that the focus is currently more on improving research opportunities. However, such improvements would become of little use to those who are currently ill. In his view, care must first become better and then improvements in the area of research can be considered. From the healthcare researcher's point of view, tangible added value would be available to patients if they were able to find their optimal level of care promptly and consequently receive targeted treatment in a timely manner. The representative of the health insurance company and the healthcare researcher highlight the aspect that patients can receive more quality-oriented support and guidance within the healthcare system, thanks to the improved possibilities of cross-sector treatment. Moreover, doctors would be able to incorporate the expertise of another doctor into their own decisions. In the context of follow-up treatment, health data would also be directly available and promptly accessible to doctors in private practice. The pharmacy representative emphasised that pharmacies could improve medication management using the available data.

The healthcare researcher states that, considering the enhancement in care, there would likely be an increase in satisfaction among service providers and patients with the German healthcare system.

## Discussion

### Discussion of results

#### Development of the EHR

All respondents rated the development of the EHR as predominantly negative since its introduction in January 2021. One of the reasons for this is that only a very small number of people with statutory health insurance have an EHR and existing records are hardly ever used by them. In turn, the low use of the records also shows that patients do not recognise any added value in this functionality. On the one hand, the cause of this can be the fact that patients were not sufficiently involved in the type and scope of the information measures and the design of the EHR. On the other hand, the cause could also be on the patient side, them being closed to digital innovations or their insufficient individual resources for developing digital skills to cope with this change.

From the perspective of the service provider, the pharmacy representative points out that the EHR is currently not being used in pharmacies. There is also a conceptual problem with the introduction of new applications such as the e-prescription or the EHR. As an example of this, he brings up the fact that in the past, various applications were introduced at the same time. Both service providers and patients did not receive sufficient support and training. The EHR developer assesses the record itself as a functional product, which was simply unable to achieve the desired effect in the area with the objectives formulated for the record. According to this, the current status of target achievement is not because of the EHR itself, but because of a lack of communicative and informative measures. As a result, the experience gained so far and the causes identified as to why the objectives formulated for the record could not be achieved should be taken into account and be included in the future design of the further implementation procedure.

The low user numbers for the EHR in the German healthcare system and the poorly rated development of the EHR against this backdrop are due, among other things, to the choice of consent procedure. In comparison, Estonia and Denmark have at least relied on an opt-out procedure when implementing the EHR, which means that almost the entire population is provided with an EHR (3, 4). In the German healthcare system, on the other hand, an opt-in procedure was used. The patient must therefore make a conscious decision in favour of an EHR and apply for it independently. The choice of consent procedure for the EHR is therefore a relevant factor in terms of successful implementation. The low availability of EHRs and their low utilisation in the German healthcare system can therefore be explained, among other things, by the choice of consent procedure. In order to achieve the widespread availability of EHRs in the German healthcare system, it is therefore necessary to rely on an opt-out procedure.

Furthermore, with regard to the process of implementing the EHR in Denmark and Estonia, it can be seen that both countries are pursuing a stand-alone digital health strategy (4, 21). This forms the framework of a structured, holistic concept according to which the EHR was implemented. There was no digitalisation strategy for the German healthcare system until March 2023. Although the various laws enacted contained requirements and objectives for the introduction of an EHR and were intended to promote the use of the health data it contains, there was no holistic framework in the form of a strategy. The pursuit of a digitalisation strategy with its individual goals and measures serves as orientation for all representatives of stakeholders involved in a complex and lengthy process. For the implementation of an EHR in the German healthcare system, it is therefore crucial in future to be guided by the strategy defined by the Federal Ministry of Health (15). The strategy's goals and measures must be actively and gradually implemented. However, it is necessary to regularly evaluate the current status of target achievement during the course of the process. If, for example, it turns out that individual goals cannot be achieved by the specified date, they must therefore also be updated and adjusted as part of the digitalisation strategy.

### Access authorisations

The interviewees express various opinions regarding the access authorisations of different stakeholders. In particular, the interests behind the required access play a role here. Accordingly, it is decisive whether there are economic interests or interests in the public. According to the representative of the SHI-accredited physicians, the fundamental question that must be clarified is which interests exist for each actor regarding access to EHR data and what justifies the type and scope of access. Only with this understanding can the discussion about corresponding access options be conducted.

Both the representatives of statutory health insurance physicians and pharmacies and the healthcare researchers emphasise that the role of record owners has not yet been sufficiently taken into account in the discussions on access authorisations thus far. In this context, comprehensive communication and information measures towards patients would have to be implemented at the outset. As part of these measures, it should be demonstrated in a simple and understandable way how this system works and where the potential of file utilisation lies in terms of improving medical care. The aim should be to create a basis of trust and enable patients to make informed decisions based on the information provided. The representative of pharmacies is also in favour of the planned opt-out procedure, as this will allow patients who do not trust the system or do not wish to use it to retain their freedom of choice. The latter aspect in particular plays a decisive role in creating a basis of trust.

Access options for all service providers involved in the treatment are a crucial prerequisite, as only on this basis can be possible the simplified interdisciplinary and cross-sectoral exchange of data be made possible.

The opinions of the interviewees differed when it came to health insurance companies' access authorisations to EHR data. While the health insurance company representative is in favour of access options and argues that the health insurance companies can better support the insured persons in their care based on the data, the pharmacy representative rejects access options for statutory health insurance companies. From the EHR developer's point of view, the access options for health insurance companies should be regulated via an opt-in procedure, as there are no public interests involved here. As already explained, this also raises the question of the interests of the respective actor behind the corresponding demand. On the one hand, the statutory health insurance funds are pursuing the goal of improving the provision of customised care services. On the other hand, however, they also have economic interests, as the services provided can in turn be billed. The choice of an opt-in procedure for the access options of health insurance funds would therefore be a conceivable solution that would preserve the freedom of choice of the insured person. With the implementation of the EHR, its use became mandatory for all healthcare institutions in Estonia and Denmark. A debate on access authorisations comparable to that in the German healthcare system has therefore not been held in these countries. In Estonia, for example, patients are allowed to decide for themselves which service providers are authorised to view their data. The reason for the extensive discussion of access authorisations in the German healthcare system is partly due to the number of structures and players involved. Compared to Estonia and Denmark, the German healthcare system has many structures and actors that need to be taken into account when making political decisions. Bertram et al. (22) describes this aspect as an inhibiting factor with regard to the digitalisation of the healthcare system. The large number of actors in self-government significantly slows down the decision-making process in connection with existing conflicts of interest.

With regard to the release of data for research purposes, it is crucial that research institutes can access the overall data of the healthcare system in anonymised form to serve as a foundation for advancing healthcare from a scientific perspective. In this context, the EHR developer also proposes the release of data via an opt-out procedure. This regulation is one approach to generating the largest possible amount of data. However, it is questionable to what extent such regulations will be approved by patients. In both Estonia and Denmark, the release of data from the EHR for research purposes is permitted (4). However, the laws applicable in the respective countries specify that the data may only be made available for research purposes in anonymised form.

### Infrastructure and technical requirements

From the point of view of the representative of the SHI-accredited physicians, a corresponding basis for decision-making, which provides for the definition of usage scenarios for the EHR, would be missing—similar to the sub-item access authorisations. Here too, the political authorities had not fulfilled their tasks in a timely manner and to a sufficient extent. As a result, the various utilisation scenarios must first be defined. This includes the individual processes and process steps as well as the involvement of the various stakeholders. Based on this, there are requirements for the further development of the infrastructure and the associated technical requirements. In addition, all interviewees see a need to define specifications for the structure of the data. In particular, the aim is to ensure that relevant data can be retrieved by various service providers in a targeted manner and processed by the systems in a partially automated manner.

From a health insurance perspective, it is crucial that data from the outpatient sector is available promptly. In fact, this aspect is also essential for the exchange of data between the outpatient and inpatient sectors. As long as the data is not available on the same day, it becomes more difficult to react quickly to acute events. The gradual approach to the ideal of real-time availability of health and care data is one of the goals of the digitalisation strategy [(15), p. 27]. The health insurance company representative suggests switching to cloud-based systems for this purpose. However, this requires an adjustment to the regulatory framework, as the use of such systems is currently only possible to a very limited extent in the German healthcare system. This changeover would facilitate the exchange of data across disciplines and sectors and would significantly increase the speed of the system. Cloud-based systems are already in use in Denmark and Estonia. In Denmark, for example, data from the individual databases of hospitals and general practitioners is transmitted to the national health portal sundhed.dk and can then be accessed by the various service providers and patients (3). Estonia also has a national health information exchange network called the Estonian National Health Information Service. Similar to Denmark, data is passed on to the health information portal by the service providers (4). The use of these systems is associated with a number of advantages. Among other things, they offer a high degree of flexibility and nationwide access options, as the data can be accessed by authorised persons at any time and from any location as long as there is an internet connection (3). The use of cloud-based systems in the German healthcare system could improve the timely availability of health data in the future.

From a service provider perspective, appropriate lead times and training for future users must be guaranteed when introducing new applications. This is a task for the health policy authorities and, according to the pharmacy representative, has not been sufficiently taken into account in the past. The latter point also highlights the conceptual problems in the German healthcare system when introducing new applications. From the healthcare researcher's point of view, it is crucial, especially when it comes to infrastructures, to involve the user stakeholders and to organise the further development in such a way that they experience added value as a result.

### Data protection and information security

The EHR developer and the representative of the health insurance fund emphasise that the current design of the registration process would represent a barrier to access for insured persons and that a more user-friendly solution must be found here. In contrast, the representative of the statutory health insurance physicians emphasised that data protection requirements should not be put on the back burner. Compliance with data protection requirements is a prerequisite for handling of health data. Similar to the issue of access authorisations, a conflict of interest between various stakeholders can be identified here. The design of the registration process is particularly crucial for the statutory health insurance funds, as this has a significant influence on the number of users of the corresponding EHR checkout app. For the care itself, the design of the registration process plays a less decisive role, as the EPR, if the patient does not object as part of an opt-out procedure, is in the system as a pure service provider file and can be used by the service providers in the context of treatment. For the representative of the statutory health insurance fund, the focus is on increasing the number of users of the health insurance app on the part of the insured. In contrast, the representative of the statutory health insurance physicians prioritises the protection of doctor-patient confidentiality.

The pharmacies' representative mentiones the requirement that patients must be able to exercise their right to self-determination when using the EHR. According to the BfDI, the current design of the EHR for access management violates the GDPR, especially for people who do not have their own device or do not want to use one. These people would become restricted in their patient sovereignty and would not be able to exercise their rights to self-determination (23). It is important to establish regulations for this group of people that enable the uncomplicated use of a representative. In addition, from the perspective of pharmacists and statutory health insurance physicians, data protection and information security must become a matter of agreement with the BfDI. As announced when the Federal Ministry of Health published its digitalisation strategy, the BfDI's right of co-determination is to be changed from agreement to consultation.

With regard to data protection and information security, the requirements of the GDPR apply in Germany, Estonia and Denmark. However, it is up to each individual country to organise the corresponding measures to ensure that the requirements of the GDPR are met. With regard to the number of existing laws and the requirements formulated therein, the German healthcare system has a comparatively strict framework in terms of data protection and information security requirements. In addition, the requirements and interests of various stakeholders must be taken into account in Germany with regard to this topic. According to former Federal Data Protection Commissioner Peter Schaar, this federal system would secure nationwide healthcare provision, but at the same time make it more difficult to implement nationwide digital information structures (24). By comparison, in Estonia and Denmark, only individual institutions are entrusted with ensuring data protection requirements are met (25, 26). This makes the decision-making process much simpler. At the same time, compliance with the requirements of the GDPR ensures that the handling of health data becomes more secure.

### Patient requirements for the EHR

The healthcare researcher and the representatives of statutory health insurance physicians and pharmacies name specific requirements for the EHR that must be met from the patient's perspective. As already mentioned in the access authorisations sub-item, comprehensive information and communication measures for patients and the creation of a basis of trust are emphasised once again. Verifiable value propositions should form the basis of a foundation of trust and corresponding information and communication measures. Formulating these is a task for the political authorities. The EHR must also become user-friendly. Particularly, it should be determined how the patient side defines the criterion of user-friendliness. In this context, it must be taken into account that Germany is strongly affected by the effects of demographic change and that the population therefore has an increasing proportion of older people (27). The consequences of demographic change must be taken into account in corresponding communication and information measures by integrating the channels that older people prefer to use. As already shown in the subsection on data protection and information security, people become excluded from EHR use due to restrictions of any kind. This aspect must be viewed critically from an ethical perspective. In order to make the EHR user-friendly, the different needs of patients must be recorded and then implemented in the design of the EHR. This could be done as part of further research, as the needs of the different user groups are complex and also depend on the age group. In addition, a test phase in which a heterogeneous group of people goes through the registration process up to the actual use of the various EHR applications would be recommended.

### Needs from the service provider perspective

Only the representative of pharmacies commented on needs that exist from the perspective of service providers. Here, he mentions, among other things, the remuneration of additional time expenditure incurred by service providers through the use of the EHR. This measure could increase the acceptance of service providers to use the EHR, as it would provide financial compensation for the increased workload. In addition, the issue of liability must become regulated before the introduction to create a secure basis for action and information for the service provider. The use of e-prescriptions and electronic certificates of incapacity for work in day-to-day care has recently led to an increase in technical faults in connection with the telematics infrastructure (28). As the use of digital applications is to be further expanded in the future, it is imperative to find a regulation for dealing with technical problems or failures. The representative of the statutory health insurance physicians does not comment on needs that exist from the point of view of service providers. This could be due to the fact that, unlike the representative of the pharmacies, he did not work in healthcare himself.

### Challenges

The healthcare researcher and the representative of the health insurance company see a challenge in bringing together the various data sets and formats so that they can be analysed together. A legal basis must first be created for this, as already explained in advance. Currently, data collected at different points in the healthcare system may neither be merged nor analysed together. The basis for this is expected to become part of the announced Health Data Utilisation Act.

In addition, a regulation must be found on how to deal with such data that was not collected in the context of medical treatment and can, for example, be set by the patient themselves. The problem here is that no clear statements can be made about how valid this data is. One possible approach would be to include data collected on a patient's exercise behaviour using appropriate wearables in the treatment of diabetes or obesity. However, this data should only be used to supplement the data collected by doctors.

The representative of the health insurance company identifies another challenge in the need to discuss corresponding sanctions alongside the introduction of a reporting obligation for service providers. The concern is that this might reduce the acceptance of using the EHR among service providers. Additionally, it entails increased bureaucratic effort, and relevant authorities must take responsibility for checking compliance with the obligations outlined in this context.

### Potential

The care researcher, the representatives of health insurance company and the pharmacies emphasise the economic benefits that arise in relation to the successful implementation of the EHR. These include, in particular, cost and time savings resulting from the avoidance of incorrect treatment and duplicate examinations. These freed-up financial and time resources can ultimately be used for more effective and targeted patient care. In addition, the avoidance of incorrect treatment and duplicate examinations is also in the interests of patients.

Improvements in the areas of care and research are also mentioned. In this context, however, the representative of the SHI-accredited physicians points out that the priority should initially be on improving care. This includes ways to improve interdisciplinary and cross-sector treatment, as the data is available more quickly. During the COVID-19 pandemic, it became particularly clear how the rapid and structured exchange of health data can contribute to the early detection and thus containment of infections and improve care (29). In addition, patients can be guided through the healthcare system in a more targeted manner in order to find the optimal level of care for them in a timely manner.

At the research level, improvements can be achieved by making a very large amount of data on the health status of the German population accessible as soon as the corresponding access rights for research projects have been clarified. The findings gained here can then in turn be used to improve care. This process is also referred to by the German Advisory Council on Health and Care as a learning healthcare system (3). This opens up possibilities for developing new forms of care. In light of the expected improvements, satisfaction among service providers and patients can be expected to increase.

## Discussion of methods and limitations

As part of the discussion of methods, we would first like to emphasise that the results, particularly due to the small sample size, are to be understood as a first step towards a deeper understanding of the introduction of the EHR and cannot cover the topic in its entirety.

A qualitative study design was consciously chosen for this study, as it allows for an in-depth exploration of the different perspectives and experiences of the actors involved in the EHR implementation process. Given the complexity of the topic and the need to develop a comprehensive understanding of the challenges and opportunities associated with the implementation of the EHR, a qualitative design provided the scope for a multi-perspective investigation. Building on the more exploratory findings of this thesis, a political science model that provides an appropriate framework in terms of decision-making, conflicts of interest and lobbying influence could now be applied to gain further insights in this area. The outcomes of our paper can provide a starting point for this. To ensure the quality of this work, the study became subject to the quality criteria proposed by Lincoln et al. (17). Trustworthiness was ensured by collecting data comprehensively and over a longer period of approximately two months in the field of investigation. The quality criterion of transferability was achieved through the dense description of the people and contextual conditions studied. In this context, personal data and information on professional development in the German healthcare system became part of the interviews. According to Lincoln et al. (17), the quality criteria of reliability and confirmability should be ensured with the help of a research audit. In the context of this work, a reviewer was used as an alternative. During data evaluation using qualitative content analysis, the data was coded in several passes and the finalised category system was discussed with the reviewer in order to avoid distortion of the results by the researcher's feelings, values, interests and motives.

As the selected sample is a convenience sample and therefore not a random sample, the sample is not fully representative of the population under investigation (16). Distortions in the study results could also be due to the composition of the sample, as three of the interviewees come from the working environment of a large statutory health insurance fund in Germany. In addition, one interviewee, who was to be interviewed as a representative of the patient side, cancelled at short notice. Therefore the patient perspective can not be analysed in this study. With regard to the survey instrument used, it must be mentioned that the guidelines contain some very large and complex subject areas. When analysing the data, it must be taken into account that the respondents' answers are subjective and depend on their professional activity and thus their proximity to one of the previously defined actors. The strengths of qualitative content analysis lie in the systematic and rule-based approach, which ensures a high level of transparency in the research process (16).

## Conclusion

The current status of EHR implementation and the low utilisation of health data in the German healthcare system in this context can be attributed to a lack of legal regulations, structural problems and the timing of implementation. In order to catch up, it is essential to implement the measures and objectives set out in the digitalisation strategy promptly and with the involvement of all stakeholders. With regard to the implementation process, Germany can orientate itself on Estonia and Denmark, for example, as the EHR is implemented nationwide there and is used extensively within the healthcare systems.

With regard to the political authorities in the German healthcare system, they did not fulfil their tasks to a sufficient extent, as the basis for decision-making was lacking and the set time frame was not adhered to.

In the context of implementation, the focus must be on ensuring that the added value of the application becomes tangible for all those involved. Only with this foundation can acceptance of the introduction of new digital applications be increased. Attention should be paid to a user-friendly design for both patients and all other stakeholders.

From the perspective of the German healthcare sector, there are various changes and challenges that need to be addressed as part of the implementation process. With regard to the service provider perspective, for example, appropriate lead times should be guaranteed and liability issues clarified in advance. When it comes to the issue of access authorisations for various stakeholders to EHR data, there is still no viable basis for decision-making. The decision-making process becomes significantly more difficult in this context, as the needs of the various stakeholders must be taken into account and the interests behind the request for data access differ greatly. In order to improve the exchange of data across disciplines and sectors, a solution is needed to ensure that data becomes available in near real time. How the use of data for research purposes will develop against the background of a successful implementation depends largely on the design of the legal framework and consequently the choice of the consent procedure for data release as well as the will of the patients. The findings obtained in this thesis can be used as a basis for further investigations in order to take a more differentiated look at the individual problems that have been identified.

## Data Availability

The raw data supporting the conclusions of this article will be made available by the authors, without undue reservation.
